# Studies on Preformulation and Formulation of JIN-001 Liquisolid Tablet with Enhanced Solubility

**DOI:** 10.3390/ph15040412

**Published:** 2022-03-28

**Authors:** Han-Sol Kim, Chang-Min Kim, An-Na Jo, Joo-Eun Kim

**Affiliations:** 1 Department of Pharmaceutical Engineering, Catholic University of Daegu, Hayang-ro 13-13, Gyeongsan-si 38430, Korea; nice604@naver.com (H.-S.K.); vhvjdi5555@naver.com (C.-M.K.); 2 J INTS BIO, 49 Achasan-ro 17-gil, Seongdong-gu, Seoul 04799, Korea; anna.jo@jintsbio.com

**Keywords:** JIN-001, heat shock protein 90 inhibitor, capryol 90, colloidal silicon dioxide, liquisolid, solubility, pharmacokinetic study

## Abstract

This study aimed to develop a heat shock protein 90 (Hsp90) inhibitor liquisolid tablet with improved solubility to overcome low bioavailability issues. As an active pharmaceutical ingredient (API), JIN-001, a novel Hsp90 inhibitor, was reported to have substantial in vitro antiproliferative and in vivo antitumor activity; however, JIN-001 was a crystalline solid with very low solubility in an aqueous solution, and therefore, Capryol 90, which has excellent solubilization ability, was selected as an optimal liquid vehicle based on solubility studies. JIN-001 liquisolid (JLS) powder was successfully prepared by dissolving JIN-001 in Capryol 90 and mixing colloidal silicon dioxide (CSD) used as an oil adsorption agent. The prepared JLS was confirmed to be amorphous. Based on the result of the solubility test of JLS, compared to JIN-001, the solubility of the former was significantly improved in all solvents regardless of pH. JLS tablets were prepared through wet granulation using JIN-001 and stable excipients based on the compatibility test. The developed JLS tablet significantly increased the drug release rate in all tested solutions; however, the liquisolid method had no significant effect on bioavailability in the pharmacokinetics study in beagle dogs. In conclusion, the liquisolid system influenced the solubility and dissolution rate of JIN-001.

## 1. Introduction

Heat shock protein 90 (Hsp90) is a molecular chaperone involved in the folding, stabilization, and activation of client proteins and the formation and degradation of transcription complexes [1]. Hsp90 plays a key role in cell growth, proliferation, and survival by regulating the activities of various signaling substances, transcriptional regulatory factors, and kinases under normal and stress conditions [2,3]. The client proteins of Hsp90 include Her2/ErbB2, v-Src, Hif-1a, Raf-1, Akt, hTERT, etc., which are involved in tumor survival and growth, and whose expressions are upregulated in cancer cells [4,5,6,7,8,9]. The expression of Hsp90 is found to be 2–10 times higher in cancer cells than in normal cells [10,11]. Hsp90 inhibitors reportedly cause misfolding of client proteins, which are degraded by the proteasome, and induce cancer cell apoptosis by blocking the cancer signaling pathway [12,13]; therefore, Hsp90 has been regarded as a promising target for cancer treatment, and various Hsp90 inhibitors have been developed and actively studied [14,15,16].

JIN-001 is a novel Hsp90 inhibitor with ring-opened dihydroxybenzamide scaffold [17]. In previous studies, JIN-001 was found to have substantial in vitro antiproliferative and in vivo antitumor activity [17]. Orally administered JIN-001 at 50 mg/kg dose exhibited a tumor growth inhibition rate of 46.9%, determined using human A549 lung cancer xenografts [17]. In particular, when the combination of afatinib and JIN-001 was administered orally, the tumor growth was delayed by 72% in the lung H1975 xenografts model [17]. In addition, JIN-001 did not show visual toxicity, a known side effect of Hsp90 inhibitors, observed using 661W photoreceptor cell lines [17]; therefore, JIN-001 has a high potential for lung cancer treatment with strong anticancer activity and safety.

Here, we confirmed that JIN-001 is a crystalline material with very low water solubility, which could lead to bioavailability issues during the design of oral dosage forms [18]. Since the absorption of poorly soluble drugs in the gastrointestinal tract is limited, the bioavailability of oral drug formulations is affected, thereby posing a major challenge to formulation scientists [19]. This is particularly applicable for drugs classified as BCS class II in the US Food and Drug Administration’s Biopharmaceutical Classification System; the dissolution rate represents the rate-determining step of the absorption process [20]. Several methods have been studied to improve solubility of poorly soluble drugs. Techniques such as drug particle size reduction, nanosuspension, salt formation, solid dispersion, prodrugs, and self-emulsifying drug delivery systems have been studied [21,22,23,24,25,26].

In the case of crystalline compounds, solid dispersion and liquisolid systems can be considered for improving the solubility of drugs via a relatively simple method that involves conversion to amorphous form [27]. Solid dispersion is a technique for dispersing drug particles in a solid aqueous matrix [28]. The drug is diffused in an aqueous matrix, present in an amorphous state with small particle size and large surface area to improve solubility and bioavailability [29]. Solid dispersions are generally prepared by solvent evaporation and melting [30,31], and have been actively studied over the past few decades, where extensive expertise has been developed to aid formulation, while its commercial application is limited [32]. This could be because of conversion to a crystalline form, challenges in expanding to an industrial scale, physicochemical stability of drugs, and expensive manufacturing equipment [33].

The liquisolid technology is being evaluated as a promising alternative approach to improve the solubility of poorly soluble drugs [34]. This technique involves dissolving a poorly soluble drug in a non-volatile liquid vehicle and further converting it into powdered form with improved flowability and compressibility by mixing a carrier and a coating material with high surface porosity [35]. The drug remains solubilized and dispersed in the carrier, where its surface area and wetting properties are increased significantly [36]. Finally, the drug can be formulated in the form of pellets or tablets with improved solubility and bioavailability [37,38]. It has been reported that solubility and bioavailability of various drugs has improved using liquisolid technology [39,40,41]. The advantage of the liquisolid system compared to other solubilization strategies is that it can be scaled for mass production and commercial application readily due to the simplicity of the manufacturing process and low production cost [42].

In this study, we decided to investigate the effect of the liquisolid method on solubility in the development of JIN-001 tablets. Drug-likeness was evaluated by examining the physicochemical properties of JIN-001–a new chemical entity. In addition, to maximize the oral absorption of JIN-001, a poorly soluble drug, an optimal liquid vehicle for solubilization of JIN-001 was selected. JIN-001 liquisolid (JLS) powder was prepared using the selected liquid vehicle, active pharmaceutical ingredient (API), and colloidal silicon dioxide (CSD), an oil adsorption base. The physicochemical properties of the prepared JLS were confirmed through scanning electron microscopy (SEM), differential scanning calorimetry (DSC), powder X-ray diffraction (PXRD) analysis, and solubility test. JLS was formulated in a tablet form using excipients with verified stability. The dissolution patterns were compared by conducting a dissolution test against other tablets manufactured using various compositions, such as tablets without solubilizer or tablets containing SLS, Poloxamer or Gelucire. The bioavailability of JLS was evaluated through pharmacokinetic studies in beagle dogs. The functionality of the JLS tablet was confirmed from the convenience of the manufacturing process. The aim of this study was to propose a solid formulation for oral administration of JIN-001 with a simple manufacturing method and improved solubility using liquisolid technology.

## 2. Results and Discussion

### 2.1. Physicochemical Properties of JIN-001 by Experimental Analysis

Table 1 summarizes the experimental results of the physicochemical properties of JIN-001. JIN-001 is a white powder and showed a sharp peak in the range of 10–40° 2θ during PXRD analysis, confirming that it is a crystalline material. In the DSC analysis, an endothermic peak in the range of 171.95–190.83 °C was observed, and the melting point measured was found to be 180.58 °C. In the TGA analysis, JIN-001 was anticipated to be unstable, as the mass loss occurred above its melting point of 180 °C. Although there was a significant difference from the melting point predicted using ChemDraw software (435.44 °C), it was suggested that the stability of compounds during the drying process after wet granulation process might not be affected.

To evaluate the hygroscopicity of JIN-001, the loss on drying (LOD) of the drug was measured and the drug was subsequently stored under various humidity conditions for 1 week to measure the equilibrium moisture content. LOD of JIN-001 was found to be 0%, and no increase in the equilibrium moisture content in all humidity conditions was observed; therefore, JIN-001 is classified as non-hygroscopic (Class I) as per the hygroscopicity classification system.

Good flow of the powder is an important factor for producing uniform tablets. Generally, when the CI value is ≥10 to ≤31 and the HR value is ≥1.00 to ≤1.45, it is estimated to have appropriate flowability for tablet production [43]. The CI of JIN-001 is 16.0%, and HR is calculated as 1.19, confirming good flowability.

**Figure 1:**
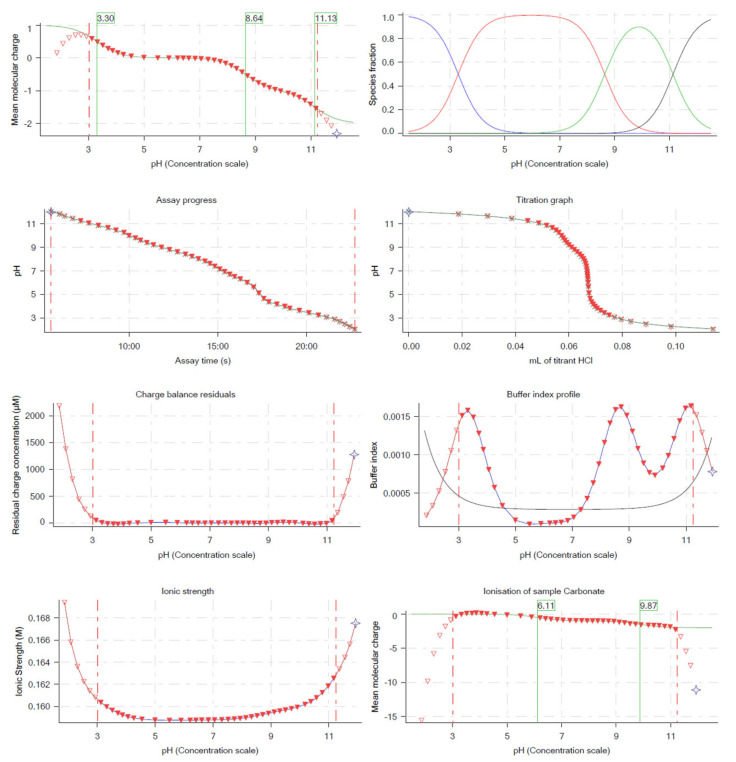
pH−metric pKa measurements of JIN-001 with Sirius T3.

To measure the pKa of JIN-001, potentiometric acid-base titration was performed to confirm the ionic form of JIN-001 depending on its pH. From Figure 1, it can be seen that the monovalent cation of JIN-001 is dominant at pH 1–3. The base pKa at which the ratio of cationic species to neutral species is 50% was 3.30. The neutral species of JIN-001 were dominant in the pH range of 3.30–8.86. After pH 8.86, the anionic species of JIN-001 were predominant, and the acid pKa was measured to be 8.86 and 11.13. This implies that JIN-001 is easily ionized at a low pH of 3.30 or less and a high pH of 8.86 and 11.13 or more, according to the Henderson–Hasselbalch equation. From these results, we concluded that JIN-001 could mainly be absorbed in the duodenum and upper small intestine environment (pH 4–7) in its neutral state [44].

Log D is the distribution coefficient between water and octanol that varies depending on the change in the pH, which is widely used to measure the lipophilicity of ionizable compounds [45]. As seen in Table 1, Log D of JIN-001 was found to be in the range of 1–3.23, estimated at physiologically relevant pH 1.0–8.0; moreover, Log P of JIN-001 was measured to be 3.32. These results suggest that JIN-001 has an appropriate volume of distribution and oral absorption. From these results, it was concluded that JIN-001 possesses physicochemical properties suitable for oral administration.

As the ChemDraw software only provides approximate values, the results are considered inaccurate because these are only predictions on the physical and chemical properties obtained through chemical structures. From the start, the physicochemical results from the ChemDraw software were unreliable; the intent of using the software was only to observe the prediction values; therefore, actual experiments were conducted to analyze and evaluate the physicochemical properties of the new drug substance. Following the confirmation of properties of the new drug substance using the prediction values obtained from the software, we were able to plan the formulation and conduct a physical experiment on the actual value.

The determination of drug-likeness is a very important. Several new drug substances have limitations on their physicochemical properties (such as solubility and permeability) that were observed during the development process of the final drug product. Additionally, these new drug substances have limitations in overcoming all of these through general formulation research; therefore, accurate understanding of the drug-likeness through preformulation will help in strategic direction of the formulation research for the final drug development. Through the determination of drug-likeness, information that is relevant in the formulation development process, such as absorption site and solubility requirements, can be obtained. Furthermore, we believe that it is a very important indicator because it provides a rough idea of how physical and chemical stability and bioavailability are predicted.

### 2.2. Solubility Studies

The solubility of drug substances remains a crucial physicochemical property affecting drug absorption. To understand the dissolution behavior of JIN-001, apparent solubility and equilibrium solubility tests were performed in water, organic solvents, and buffers of various pH ranges (Table 2). The apparent solubility test results of JIN-001 were classified according to the USP solubility criteria. JIN-001 was evaluated to be freely soluble with a solubility of 100 mg/mL or more in methanol and ethanol while being sparingly soluble in acetonitrile, indicating that the solubility of JIN-001 was relatively high in organic solvents; however, it was reported as practically insoluble with a solubility of 0.1 mg/mL or less in water and other buffers except for pH 1.2 and pH 12.0 buffers, where it was regarded as slightly soluble in the latter two buffers. Based on these results, it was confirmed that JIN-001 is a poorly soluble drug with very low solubility in water and buffer solution.

As summarized in Table 2, the equilibrium solubility assessment of JIN-001 performed in various solvents decreased in the following order: methanol (140.02 mg/mL), ethanol (68.27 mg/mL), and acetonitrile (13.56 mg/mL). This confirmed that the compound could dissolve well in organic solvents. Additionally, the solubility of drugs in organic solvents can be used to develop analytical methods, such as content assay and impurity tests. These solubility studies can also determine the type of organic solvent that can be used in manufacturing procedures such as wet granulation and how much organic solvent can be used in the manufacture of granules. JIN-001 had a very low solubility of 0.04 mg/mL in water, while that in the pH 1.2 buffer was 9.01 mg/mL, confirming that it has a relatively high solubility in the latter; however, it exhibited solubility of 0.70 mg/mL at pH 2.0, and that of 0.10 mg/mL or less in a buffer solution at pH range 3–11, and moreover, a solubility of 0.94 mg/mL was observed in the pH 12.0 buffer. Combining these results, it can be concluded that JIN-001 is a poorly soluble drug with very low solubility in an aqueous solution.

### 2.3. Screening of Non-Volatile Liquid Vehicle

The liquid vehicle used in the liquisolid system should be selected from a list of non-volatile organic solvent that is safe for oral administration, is inactive, and has non-viscous properties [34]. In this study, oil that could excellently solubilize JIN-001 was selected as a liquid vehicle based on the oil solubility test. When the solubility of a drug in a solvent is high, a smaller amount of solid carrier is required, leading to an improved dissolution rate [34].

Table 3 summarizes the results of the apparent solubility test of JIN-001 in various oils, where JIN-001 had the highest solubility in the order of Transcutol P, Capryol 90, Lauroglycol 90, and Labrador, with the value of 10 mg/mL or more. Other oils showed a relatively low solubility of less than 6 mg/mL.

**Figure 2:**
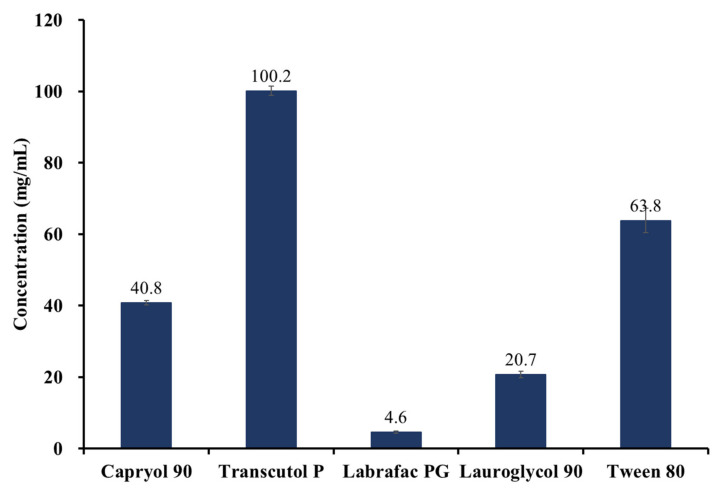
The Solubility of JIN-001 in various oils (*n* = 3).

The results of the oil solubility study are shown in Figure 2. The results showed that Transcutol P exhibited the highest solubility (100.2 ± 1.3 mg/mL), Tween 80 (63.8 ± 3.4 mg/mL), Capryol 90 (40.8 ± 0.7 mg/mL), Lauglycol 90 (20.7 ± 0.9 mg/mL), and labrafac PG (4.6 ± 0.3 mg/mL), all described in the order of decreased solubility. According to the FDA Inactive Ingredient Search for Approved Drug Products, Transcutol P is mainly used for topical purposes and has not been approved for oral administration. In the case of Tween 80, the viscosity was 555.5 ± 48.1 cP when measured with a viscometer (DV2T, Brookfield, WI, USA), confirming that it could cause problems in adsorption and mixing solid carriers, hence was excluded from the list of liquid vehicles. Following this, a relatively high soluble compared to the remaining oils, Capryol 90, was selected as the optimal liquid vehicle to increase the solubility of the API.

### 2.4. Compatibility Studies between API and Excipients

**Figure 3:**
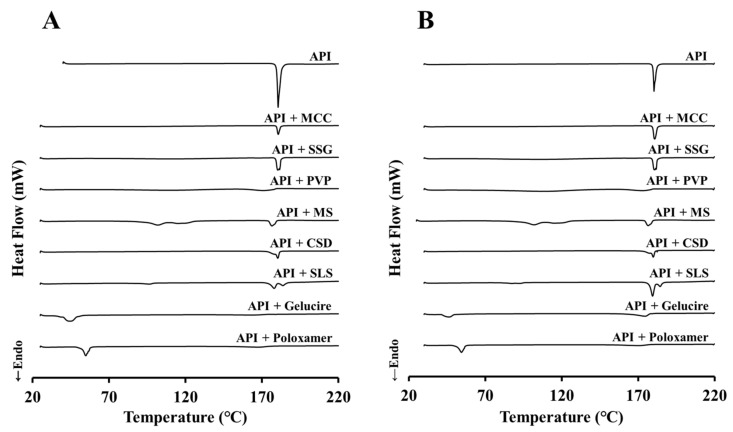
The results of compatibility studies via DSC. (**A**) Initial; (**B**) 4w, 40 ± 2 °C/75 ± 5% RH.

JIN-001 and excipients were mixed in a ratio of 1:1 (*w*/*w*) to conduct compatibility tests. Figure 3 shows the DSC analysis results of the API/excipient mixture, showing the initial results (Figure 3A) and the analysis results after storage for 4 weeks at 40 ± 2 °C/75 ± 5% RH (Figure 3B). In the case of JIN-001, an endothermic peak was exhibited at about 180 °C, and no significant peak change was observed at the 4-week result. In most of the mixtures investigated, no considerable change was observed between the initial and the 4-week results; however, the endothermic peak of the API was weak in the PVP mixture and was not observed in the Gelucire mixture and Poloxamer mixture. This shows the possibility that the API was included in PVP, Gelucire, and Poloxamer or that the crystal form was changed to amorphous.

In order to understand the amounts of impurities generated by the interaction of the API and excipients, impurity analysis was conducted through HPLC. According to the ICH Q3B (R2) guideline, the impurity of the standard was set at 0.5%, which is the optimum value for formulations with a maximum daily dose of 10–100 mg; however, the JIN-001 drug substance contains about 0.4% of impurities, set based on a 0.5% increase compared to the initial. JIN-001 initially contained 0.39% of impurities, but after 4 weeks of storage at 40 ± 2 °C/75 ± 5% RH, the value was increased to 0.42%, thereby confirming to be a highly stable material (Table 4). In addition, in most of the API/excipient mixtures, low impurities below the standard were generated at the initial and 4 weeks; however, it was confirmed that more than 5% of the impurities were rapidly generated in the sodium stearyl fumarate (SSF) mixture. Among the excipients used (citric acid and calcium hydroxide) as pH control agents, only calcium hydroxide, an alkylating agent, showed a higher percentage of impurities than the standard value. Based on these results, it can be concluded that the excipients used in the compatibility test are stable with no interaction with the API, except for SSF and calcium hydroxide.

### 2.5. Physicochemical Properties of JLS

#### 2.5.1. Morphology

**Figure 4:**
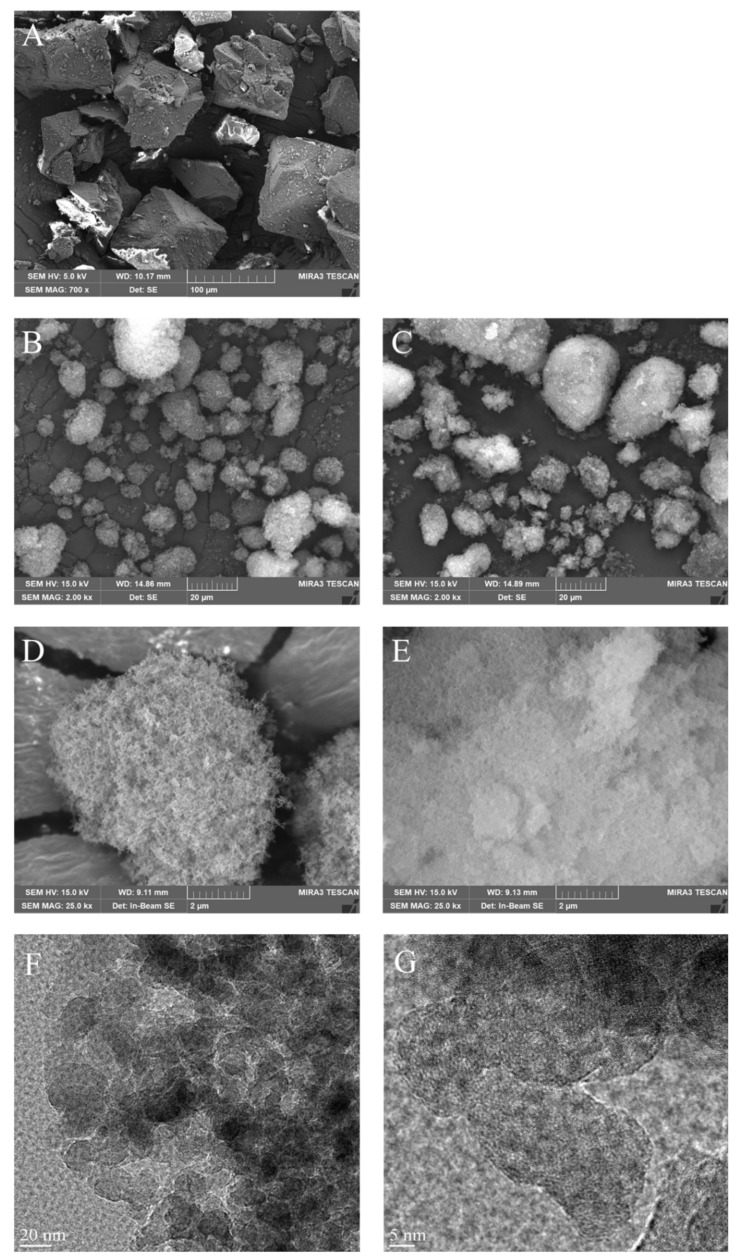
Scanning electron micrographs showing the morphology of (**A**) JIN-001 at 700× magnification (Scale bar = 100 μm); (**B**) CSD at 2000× magnification (Scale bar = 20 μm); (**C**) JLS at 2000× magnification (Scale bar = 20 μm); (**D**) CSD at 25,000× magnification (Scale bar = 2 μm); (**E**) JLS at 25,000× magnification (Scale bar = 2 μm). Transmittance electron microscopy (TEM) micrographs showing the morphology of (**F**) JLS at 105,000× magnification (Scale bar = 20 nm); (**G**) JLS at 290,000× magnification (Scale bar = 5 nm).

To confirm the properties of JLS powder, scanning and transmission electron micrographs (SEM and TEM) of JIN-001, CSD, and JLS were obtained and compared (Figure 4). After observing JIN-001 at a magnification of 700 times, it was confirmed to be a crystalline material with an angular shape (Figure 4A). CSD appeared as particles with internal cavities (Figure 4B,D). The shape of the JLS prepared by using CSD was very similar to that of CSD but with filled internal cavities (Figure 4C,E). These results confirmed that the API was well adsorbed to the CSD, and there was no aggregation phenomenon. As shown in Figure 4F,G, we elucidated the internal structure through accurate complementary analysis by transmission electron microscopy (TEM). The shape and structure of CSD and JLS were analyzed using signals obtained by passing an electron beam, accelerated using an electron gun, through a sample with a thickness of 100 nm or less. For the analysis, FE-TEM (Titan G2 ChemiSTEM Cs Probe, FEI Company, Hillsboro, OR, USA) with an acceleration voltage of 20 kV was used. We confirmed the internal structure through TEM and confirmed that the absorption capacity of the JLS system can be seen more clearly. As a result, it was confirmed that the JLS system has Capryol 90 oil in which the active pharmaceutical ingredient is dissolved in the CSD.

#### 2.5.2. Thermodynamic Analysis and Crystallinity

**Figure 5:**
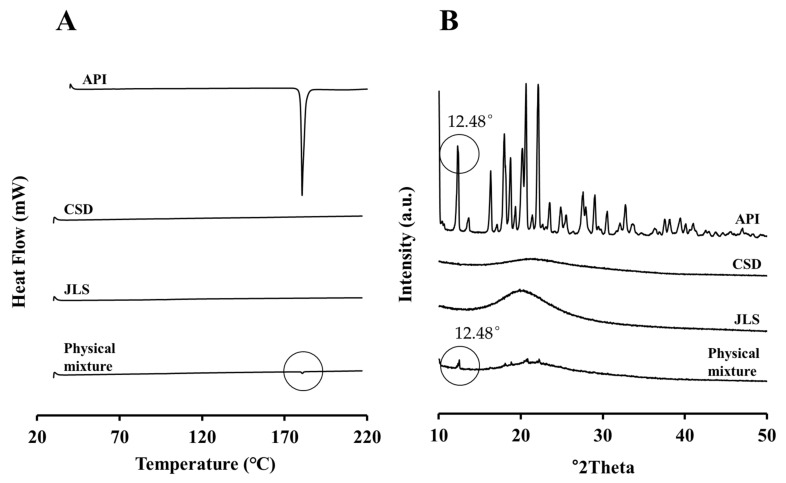
Physicochemical properties of the interaction between JIN-001 and JLS. (**A**) DSC analysis; (**B**) Powder X-ray diffraction (PXRD) analysis.

To evaluate the crystallinity of JLS powder, DSC and PXRD analyses were performed on JIN-001, CSD, their physical mixture, and JLS (Figure 5). Figure 5A depicts the API peak in the physical mixture of API and CSD due to DSC analysis; however, the peak of the API was not observed in JLS prepared as liquisolid using Capryol 90 and CSD. Similarly, in the PXRD analysis of Figure 5B, a peak at 12.48°2θ was observed in the same way as the peak of the API in the physical mixture. This indicates that JIN-001 maintains crystallinity in the physical mixture; however, no crystalline peak of the API was observed in JLS. Based on these results, it was confirmed that JIN-001, a crystalline drug, was dispersed in JLS in an amorphous form.

#### 2.5.3. Solubility of JLS

**Figure 6:**
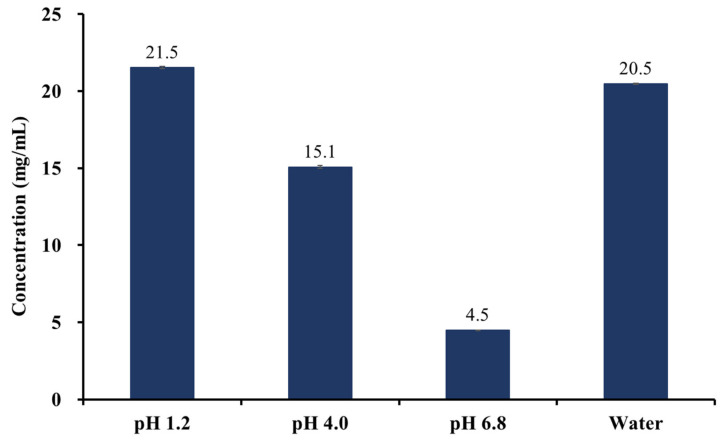
The Solubility of JLS in water, pH 1.2, pH 4.0, pH 6.8 buffer (*n* = 3).

To evaluate the solubilizing ability of the liquisolid system, the solubility of JLS in pH 1.2, 4.0, and 6.8 buffers and water was evaluated. As shown in Figure 6, the solubility of JLS was analyzed as 21.5 ± 0.1 mg/mL at pH 1.2, 15.1 ± 0.1 mg/mL at pH 4.0, 4.5 ± 0.0 mg/mL at pH 6.8, and 20.5 ± 0.0 mg/mL in water, with a solubility value of about 2.4 times, 188.8 times, 90.0 times, and 512.5 times, respectively, compared to that of JIN-001 in the same solution; therefore, it was confirmed that the liquisolid system significantly improved the solubility of JIN-001 regardless of pH. This is anticipated because JIN-001 is dispersed as an amorphous state in CSD, and the surface area increases as the particle size decrease.

#### 2.5.4. Flowability

Prior to the tableting of JIN-001 tablets, the flowability of JLS powder and the F2–6 prescription mixtures were evaluated by measuring CI and HR. As shown in Table 5, the CI value of the JLS mixture is 22.91 ± 0.60%, and the HR value is 1.30 ± 0.01, making it suitable for refining. Among the F2–6 mixtures, it was found that the flowability of the F4 mixture prepared by JLS was optimum, suggesting that other mixtures also possessed pertinent flowability needed for tablet production.

Although the JLS mixture has excellent flowability, it was confirmed that the tablets were oily when tableted alone, resulting in various defects such as capping and lamination; therefore, as in the composition of F4, by mixing MCC, PVP, SSG, and MG, it was possible to successfully manufacture JIN-001 tablets with no tablet defects and having a hardness of about 8 kp.

In addition, compared to the direct tableting method, the wet granulation method could ensure excellent flowability and content uniformity of powder. In the direct compression method, preliminary studies noted the powder was not properly supplied from the hopper in the tableting machine owing to the low flowability of the mixed powder, which resulted in variation in content uniformity of approximately 5% or more; therefore, our research team was able to obtain constant flowability and unvarying content uniformity by introducing the wet granulation method as a manufacturing method. To obtain excellent flowability, JIN-001 tablets were prepared using the wet granulation method.

In our study, it was confirmed that there were no issues in the flowability and impurity of granules and tablet manufacturing when the LOD was 2.5% or less. In the preliminary studies, when the granules were not sufficiently dried and the moisture content was high (2.5% or higher), an issue was noted in the flowability of granules, and related substances tended to increase rapidly. If the LOD was more than 2.5%, the flowability of the mixture was not good in the hopper of the tableting machine; hence, content uniformity determines the quality of tablets. When the LOD was less than 2.5%, there were no major issues on the quality of tablets. In addition, during tablet manufacturing, an LOD of 0.4% or less caused lamination phenomenon of the tablet, and an LOD of more than 2.5% caused sticking phenomenon on the tablet; moreover, as the LOD might be influence for the changes on dissolution rate pattern, it was then determined that the LOD should be at 2.5% or less.

### 2.6. In Vitro Dissolution Studies

In vitro dissolution study of JIN-001 formulation was performed in water and pH 1.2 buffer. In addition, dissolution tests were performed in pH 4.0 and 6.8 buffers with 0.5% (*w*/*v*) Tween 80 to measure and compare the API content released from the JIN-001 formulation. The F1 capsule was prepared by completely dissolving JIN-001 in the selected liquid vehicle, Capryol 90, and then filling it into a hard capsule. JIN-001 tablets were prepared with a hardness of about 8 kp in various compositions using various solubilizers. Tablets without solubilizer (F3), tablets with JLS (F4), and tablets with solubilizer (F2, F5, F6) were successfully prepared. The prepared formulation was subjected to a dissolution test for 1 h, and then the release amount of the API was analyzed through HPLC.

**Figure 7:**
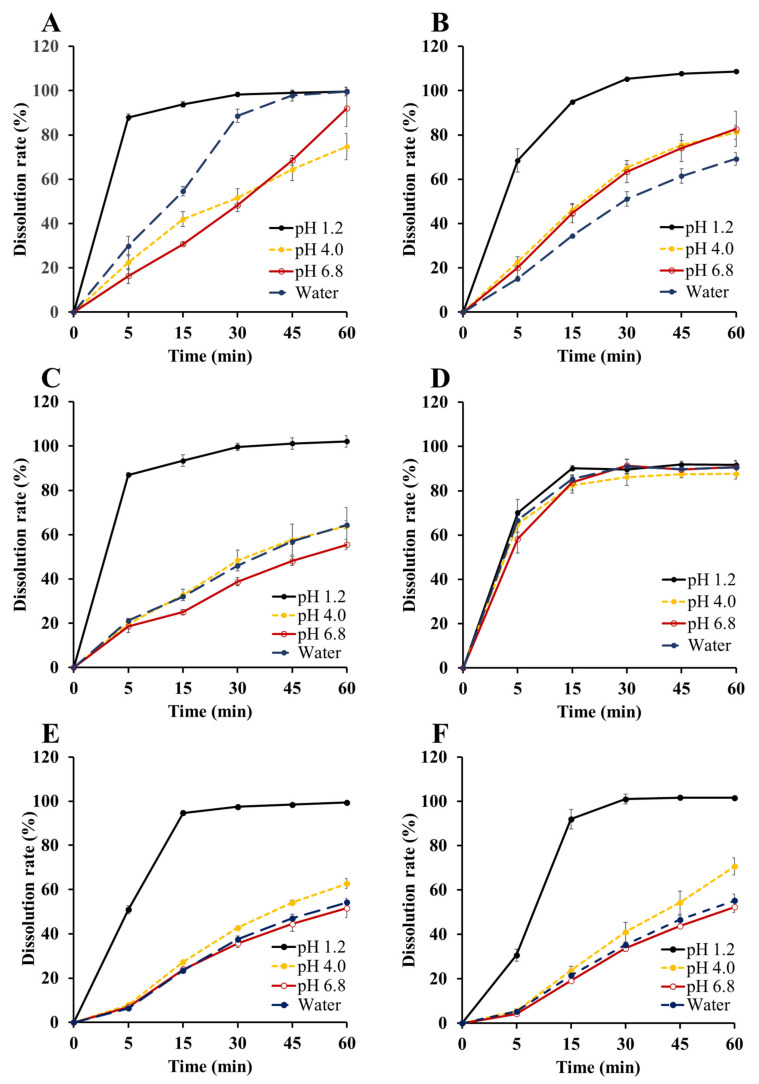
In vitro dissolution of JIN-001 formulations (*n* = 3). (**A**) JIN-001 + Capryoil 90 capsule (F1); (**B**) JIN-001 tablet with SLS (F2); (**C**) JIN-001 tablet without solubilizer (F3); (**D**) JLS tablet (F4); (**E**) JIN-001 tablet with Gelucire (F5); (**F**) JIN-001 tablet with Poloxamer (F6).

As shown in Figure 7C, the 30-min dissolution rate of the F3 tablet (without solubilizer) showed a complete dissolution rate of 99.6 ± 1.6% in the pH 1.2 solution, but 48.4 ± 4.7% and 38.7 ± 1.8% in the pH 4.0 and 6.8 solutions, respectively; moreover, a low dissolution rate of 46.0 ± 0.9% in water was observed. These results suggest that the formulation of JIN-001, a poorly soluble drug, as an oral dosage form without an appropriate solubilization strategy, may lead to low absorption and bioavailability due to the low dissolution rate of the drug in water and high pH values.

In Figure 7A, the 30-min dissolution rate of the F1 capsule (JIN-001 dissolved in Capryol 90) was 98.3 ± 1.0% in the pH 1.2 solution, 51.7 ± 4.0% in the pH 4.0 solution, 48.3 ± 2.9% in the pH 6.8 solution, and 88.6 ± 3.0% in water. F1 capsules significantly improved dissolution rate in water compared to F3 tablets. In addition, the dissolution rates of the F1 capsules at 60 min in the pH 4.0 and pH 6.8 solutions were improved by 10.9% and 36.5% compared to the F3 tablets, respectively; however, complete dissolution was not achieved in the pH 4.0 solution.

As shown in Figure 7D, the dissolution rate of F4 tablets at 30 min was 89.7 ± 1.7% in the pH 1.2 solution, 86.1 ± 3.7% in the pH 4.0 solution, 91.4 ± 2.7% in the pH 6.8 solution, and 91.0 ± 3.3% in the water. It was confirmed that the dissolution rate of the F4 tablet was 80% or more at 30 min, which is the standard dissolution rate of the immediate-release formulation among all the tested dissolution solutions. It was also confirmed that the dissolution rate was significantly increased compared to the F3 tablet in which a solubilizer was not used. Based on these results, in the case of the F4 tablet, it can be concluded that the liquisolid system improved the solubility and dissolution rate for JIN-001 in various solutions of varied pH.

As shown in Figure 7B, it was confirmed that the dissolution rate was improved even in the F2 tablet using 1% SLS. The dissolution rate of the F2 tablet was 105.3 ± 0.1% in the pH 1.2 solution, 65.2 ± 1.5% in the pH 4.0 solution, 63.4 ± 5.0% in the pH 6.8 solution, and 51.1 ± 3.2% in the water. The dissolution rate of the F2 tablet was slightly improved compared to that of F3 tablet pH 4.0 and 6.8 solutions; however, the dissolution rate at 30 min did not exceed 80%, and dissolution was not completed at 1 h.

In Figure 7E,F, the dissolution rates of the F5 tablet with Gelucire and the F6 tablet with Poloxamer were lower than those of the F3 tablet with no solubilizer. The dissolution rate of the F5 tablet was 97.6 ± 0.8% in the pH 1.2 solution, 42.8 ± 0.4% in the pH 4.0 solution, 35.7 ± 1.9% in the pH 6.8 solution, and 37.6 ± 1.4% in the water; moreover, the dissolution rate of the F6 tablet was 101.2 ± 2.2% in the pH 1.2 solution, 41.1 ± 4.5% in the pH 4.0 solution, 33.8 ± 1.3% in the pH 6.8 solution, and 35.4 ± 0.6% in the water; therefore, it was confirmed that Gelucire and Poloxamer did not improve the dissolution rate of JIN-001. Based on these results, it can be concluded that the liquisolid system is more suitable than the addition of a general solubilizer for the development of JIN-001 formulation.

### 2.7. Pharmacokinetic Studies in Beagle Dogs

**Figure 8:**
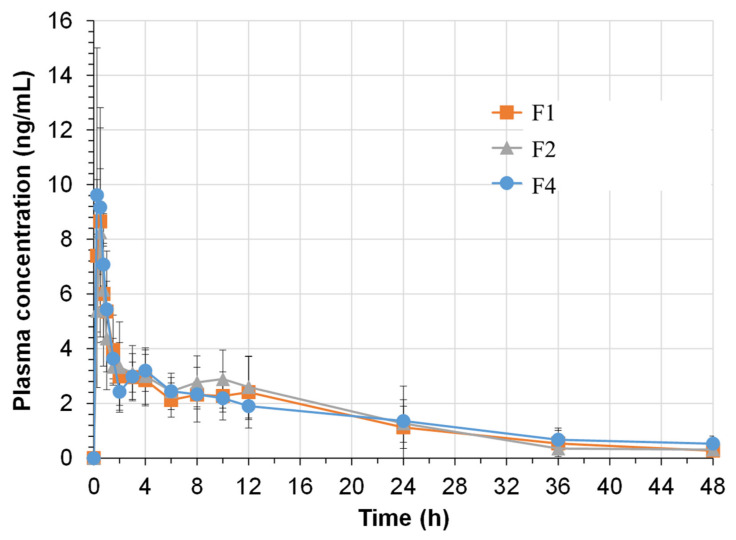
Plasma concentration-time profiles of JIN-001 following single oral administration of F1, F2 and F4 formulations (20 mg) to beagle dogs (*n* = 9).

To study the drug absorption and bioavailability of the JIN-001 formulation, the pharmacokinetic profiles of F1, F2, and F4 formulations in beagle dogs were compared. Figure 8 shows the average plasma concentration-time profiles of JIN-001 in beagle dogs. The mean of each pharmacokinetic parameter of JIN-001 evaluated in beagle dogs is summarized in Table 6. Bioavailability refers to the ratio of the amount of active drug that reaches the systemic circulation to the administered drug and is quantified and described using the area under the curve (AUC). The absorption rate of a drug is evaluated by Tmax, the time at which the concentration in the blood reaches a maximum after drug administration. Cmax is the highest concentration in the blood after drug administration. It is a parameter indicating whether sufficient absorption is achieved to produce a therapeutic response. In this study, the F4 formulation had an average Cmax of 11.29 ± 4.47 ng/mL, which was the highest among the tested formulations; however, AUC infinity was 71.25 ± 26.50 ng·h/mL, 78.48 ± 25.64 ng·h/mL, and 82.37 ± 28.79 ng·h/mL in F1, F2, and F4 formulations, respectively, and therefore, there was no significant difference in bioavailability between the formulations. The Tmax and blood half-lives of the F4 formulation were 0.47 ± 0.15 h and 14.28 ± 6.91, respectively, confirming that they were rapidly absorbed after oral administration and then slowly disappeared over 48 h. These results confirmed that the liquisolid system used in this study improved the solubility and dissolution rate of JIN-001, but did not significantly affect the bioavailability.

As shown in Figure 7, based on the dissolution results, only F4 (JLS tablet) was 100% eluted at all pH mediums. It was predicted, therefore, to show significantly different results from the liquid formulation (F1) or general tablet formulation (F2); however, the PK results in this study were not significantly different. PK results of the liquisolid tablet should have been better than those of the general tablet, but the findings showed no significant difference. Perhaps what is suspicious of these results is that, in order to create a human-like gastric environment, pentagastrin was overdose to beagle dogs to stimulate gastric acid secretion, and a PK study was conducted. As can be seen from the dissolution results in Figure 7, all formulations show the best dissolution rate in pH1.2 medium. For this reason, it is estimated that similar results may have been obtained in the PK results of all formulations. Nevertheless, the liquisolid tablet showed good results in in vitro dissolution; thus, it will surely demonstrate different results in the follow-up studies. Currently, two follow-up studies are being prepared: first, when the three formulations are administered without pentagastrin to beagle dogs; and the second is a bioavailability study by manufacturing a sustained-release gastric retention tablet.

## 3. Materials and Methods

### 3.1. Materials

JIN-001 was provided by Formosa laboratories. INC. (Taoyuan, Taiwan). Propylene glycol monocaprylate type II (Capryol 90) was purchased from Gattefosse (Lyon, France). Whereas CSD (Aerosil 200) was obtained from Evonik Industries (Essen, Germany). Each chemical was purchased from its respective company: Microcrystalline cellulose (Heweten 102, JRS Pharma, Rosenberg, Germany), Polyvinylpyrrolidone (Kollidon 25, BASF Pharma, Ludwigshafen, Germany), Hydroxypropyl Cellulose (NISSO HPC-SL, Nippon Soda Co., Ltd., Tokushima, Japan), Sodium starch glycolate (GLYCOLYS, Roquette, Lestrem, France), Sodium lauryl sulfate (Daejung Chemicals & Metals, Siheung, Korea), Magnesium stearate (Faci Asia Pacific Pty Ltd., Jurong Island, Singapore), Sodium stearyl fumarate (JRS Pharma, Rosenberg, Germany), Poloxamer (SYNPERONIC PE/F, CRODA, Singapore), Lauroyl polyoxyl-32 glycerides (GELUCIRE 44/14, Gattefosse, Lyon, France), Citric acid anhydrous (SHINWON, Eumseong, Korea), Calcium hydroxide (Spectrum, Gardena, CA, USA). Acetonitrile and methanol were obtained from DUKSAN (Ansan, Korea) in HPLC grade. Deionized water was obtained from the deionized water manufacturing equipment (Pure power I+, Human Corporation, Seoul, Korea), with a resistivity of 18.2 MΩ. All other reagents were reagent grade and were purchased commercially.

### 3.2. Physicochemical Properties of JIN-001 by Experimental Analysis

#### 3.2.1. Scanning Electron Microscopy (SEM)

The surface morphology of the drug was examined through a scanning electron microscope (MIRA3, TESCAN, Kohoutovice, Czech Republic). The accelerating voltage was set to 5.0 kV or 15.0 kV, and images were captured at magnifications of 700×, 2000×, and 25,000×. The drug was thinly coated with platinum to prevent the charge-up phenomenon (electrons accumulating on the sample surface) prior to analysis.

#### 3.2.2. Transmission Electron Microscopy

The shape and structure of CSD and JLS were analyzed using signals obtained by passing an electron beam, accelerated with an electron gun through a sample with a thickness of 100 nm or less. For the analysis, FE-TEM (Titan G2 ChemiSTEM Cs Probe, FEI Company, Hillsboro, OR, USA) with an acceleration voltage of 20 kV was used.

#### 3.2.3. Powder-X-Ray Diffraction (PXRD)

The crystal structure of the drug was evaluated using a powder X-ray diffraction system (MPD for bulk, DIATOME, Nidau, Switzerland) equipped with a PIXcel3D detector. The voltage and current were set to 40 kV and 30 mA, and the powder sample was analyzed at a 2θ angle of 7–50° with Ni-filtered Cu-Kα radiation (λ = 1.5406 Å).

#### 3.2.4. Differential Scanning Calorimetry (DSC)

To determine the thermal behavior of the drug, the drug was analyzed using a differential scanning calorimeter (DSC Q2000, TA Instruments, New Castle, DE, USA). About 2 mg of the drug was added to an aluminum pan to create a disk, and heated under nitrogen gas at a temperature range of 25–220 °C and a heating rate of 10 °C/min. The temperature range in which the sample melted and showed an endothermic peak was considered as the melting point.

#### 3.2.5. Thermogravimetric Analysis (TGA)

The thermal stability of JIN-001 was evaluated through thermogravimetric analysis (Discovery TGA, TA Instruments, New Castle, DE, USA). About 8 mg of a drug sample was heated from 40 °C to 300 °C at a heating rate of 10 °C/min under nitrogen gas to measure the decrease in drug weight with temperature.

#### 3.2.6. pKa, Log P, Log D

The pKa, Log P, and Log D of JIN-001 were evaluated according to potentiometric acid-base titration using a Sirius T3 (Pion Inc, Riverside, UK). For pKa, the pH when the ionized state and the neutral state are the same at a 50% ratio from the titration curve, estimated by acid-base titration in the pH range of 1.0–12.0 was measured. Log P and Log D were measured for partition coefficient and distribution coefficient by acid-base titration using the same method as pKa in a dual-phase solvent system of octanol and water.

#### 3.2.7. Hygroscopicity

The hygroscopicity of JIN-001 was classified according to the hygroscopicity classification system by measuring the equilibrium moisture content of the drug under specific humidity conditions [46]. First, the LOD of the drug was evaluated by drying it at 105 °C for 20 min using a halogen moisture analyzer (HB43-S, METTLER TOLEDO, Greifensee, Switzerland). After the drug was exposed to constant temperature (25 ± 2 °C) and humidity (40, 60, 75, 93 ± 5% RH) in a sealed desiccator for 1 week, the change in mass was measured. The water content (P) of the drug was calculated applying the Equation (1) with the initial mass (W) of the drug, the LOD (A), and the change in the mass of the drug (B).

(1) $$P=\frac{\left(W\times A/100\right)+B\times 100}{W-\left(W\times A/100\right)}$$

The equilibrium moisture content of the drug was calculated through Equation (2).

(2) $$\mathrm{EMC}=\left(\frac{P}{P+100}\right)\times 100$$

#### 3.2.8. Flowability

The flowability of the powder was evaluated by Carr’s index (CI) and Hausner ratio (HR). JIN-001 (10 g) was placed in a 20 mL graduated cylinder, and the volume was measured to calculate the bulk density (g/mL). Further, the tapped density (g/mL) was calculated by tapping and measuring the volume at a rate of 60 times per minute until the volume no longer decreased. Flowability was evaluated by obtaining CI and HR using the Equations (3) and (4).

(3) $${\mathrm{Carr}}^{\prime }s\;\mathrm{Index}\;\left(\%\right)=\frac{\mathrm{TD}-\mathrm{BD}}{\mathrm{TD}}\times 100$$

(4) $$\mathrm{Hausner}\;\mathrm{ratio}=\frac{\mathrm{TD}}{\mathrm{BD}}$$

### 3.3. Solubility Studies

The solubility of JIN-001 was evaluated by the apparent solubility test method and the equilibrium solubility test method in water, ethanol, methanol, acetonitrile, oils, and various buffers ranging from pH 1.2 to 12.0 [47,48].

A quantity of 10 mg of JIN-001 was precisely weighed and placed in each of 33 clear glass vial. Next, 200 μL of each solvent was slowly added to the vial and stirred at 800 rpm using a magnetic stirrer. The solvent was added until the drug was all dissolved and particles were not seen with the naked eye, and the apparent solubility was calculated depending on the amount of the added solvent.

Based on the results of the apparent solubility test, a solution containing the respective solvent and excess drug was prepared and placed in each clear glass vial. Using a magnetic stirrer, the mixture was stirred at 800 rpm for 24 h. The solution of 1.5 mL was collected and centrifuged at 10,000 rpm for 15 min. The supernatant was collected, filtered through a 0.45 μm RC syringe filter, and diluted with acetonitrile or methanol. This solution was analyzed using the HPLC method described in Section 3.8 to evaluate equilibrium solubility.

### 3.4. Compatibility Studies between API and Excipients

The compatibility between JIN-001 and excipients was evaluated by analyzing the appearance, DSC, and impurities in the drug/excipient mixture. JIN-001 was mixed with various excipients in a ratio of 1:1 (*w*/*w*) and placed in each clear glass vial. For the JIN-001/Capryol 90 sample, a mixture of JIN-001 and Capryol 90 in a ratio of 1:25 (*w*/*w*) was placed in a vial. Samples were stored for 4 weeks under two storage conditions (25 ± 2 °C/60 ± 5% RH, 40 ± 2 °C/75 ± 5% RH) in a light-blocking stability chamber. Samples were drawn at scheduled intervals and subjected to appearance, DSC (Section 3.2.3), and impurity analysis (Section 3.8).

### 3.5. Preparation of JOS

Capryol 90 (12.5 g) was precisely weighed and placed in a clear glass beaker. Next, 0.5 g of JIN-001 was added and completely dissolved by sonication for 30 min to prepare a transparent liquid mixture. Subsequently, 130 mg of this mixture was filled in a hard capsule to prepare an F1 formulation.

A total of 13 g of a liquid mixture of JIN-001 and Capryol 90 and 6 g of CSD were placed in a beaker and stirred with the impeller of a stirrer (HS-120A, DIHAN Scientific Co., Ltd., Seoul, Korea). After drying at 60 °C for 1 h, JIN-001 liquisolid (JLS) was prepared. In contrast, a physical mixture was prepared by simply mixing API and CSD at 1:12 (*w*/*w*).

### 3.6. Preparation of JIN-001 Tablets

JIN-001 tablets were manufactured through wet granulation for excellent flowability of powder mixture and content uniformity, and excipients with stable compatibility with API were selected through a compatibility test. Specifically, JIN-001, microcrystalline cellulose (MCC), polyvinylpyrrolidone (PVP), and sodium starch glycolate (SSG) were mixed to obtain the respective compositions shown in Table 7. In the case of F2, F3, and F4, wet granules were prepared by adding deionized water to the mixture [49]. For F5 and F6, wet granules were prepared by using a solution obtained by dissolving Gelucire or Poloxamer in ethanol as a binding solution, respectively. The wet granules were dried at 60 °C in a drying oven until the LOD was less than 2.5%. The dried granules were sieved through an 18-mesh sieve. Magnesium stearate was sieved through a 40-mesh sieve and finally mixed. F2 granules and other granules were compressed with a rotary tablet press machine (PR-LM08, PTK, Gimpo, Korea) using a 7 mm round punch or a 16 × 8 mm oval punch.

### 3.7. In vitro Dissolution Studies

In vitro dissolution test of a formulation containing JIN-001 was performed according to USP <711> Dissolution Apparatus 2 (paddle apparatus) using a dissolution tester (KDT1200, Kukje Engineering, Goyang, Korea). A sinker was used for the F1 formulation. For the dissolution test solution, 900 mL each of pH 1.2 buffer and water were used. Additionally, 900 mL each of pH 4.0 and 6.8 buffer with 0.5% (*w*/*v*) Tween 80 were used. The temperature of solutions was maintained at 37.0 ± 0.5 °C. The speed of the paddle was set at 50 rpm. At 5, 15, 30, 45, and 60 min after starting dissolution, 3 mL of the sample was collected and filtered through a 0.45 μm RC syringe filter. The concentration of JIN-001 in the sample was analyzed according to the HPLC content analysis method in Section 3.8.

### 3.8. HPLC Analysis

The content and impurities of JIN-001 in the sample were analyzed using an HPLC system (Agilent 1260 Infinity II LC System, Agilent Technologies, Santa Clara, CA, USA) equipped with a UV detector. An Agilent C18 column (4.6 × 150 mm, 3.5 μm) was used as a stationary phase. For the mobile phase, 0.5% (*v*/*v*) triethylamine buffer (a solution adjusted to pH 7.0 with 85% phosphoric acid) and acetonitrile were used, where the composition was changed by gradient elution. In the dissolution test, acetonitrile was set to 50% at 0 min, 95% at 5 min, 95% at 8 min, 50% at 10 min, and 50% at 20 min. In the impurity test, acetonitrile was set to 35% at 0 min, 65% at 25 min, 90% at 35 min, 90% at 45 min, 35% at 50 min, and 35% at 60 min. Prior to analysis, the mobile phase was filtered through a 0.45 μm membrane filter and degassed through sonication. The measurement wavelength was set to 280 nm, and the column temperature and flow rate were maintained at 40 °C and 0.3 mL/min, respectively. For the solubility and dissolution test of the API, the sample injection amount was 10 µL and the total run time was 20 min, respectively. The impurity test was analyzed for 60 min with a sample injection volume of 20 µL.

### 3.9. Pharmacokinetic Studies in Beagle Dogs

The pharmacokinetic test of the JIN-001 formulations in beagle dogs was conducted at KNOTUS CO., Ltd. (Incheon, Korea) and was approved by the ethics committee (KNOTUS-IACUC 21-KE-477). Nine male beagle dogs aged between 8 to 9 months, provided by Saeronbio Inc. (Uiwang-si, Korea), were used in this study. The control group was the F1 formulation in which the API was dissolved in Capryol 90, and the test group was the F2 formulation containing SLS and the F4 formulation with JLS, both set up in three groups, consisting of three animals each. After maintaining the fasting state for more than 16 h, pentagastrin was intramuscularly administered at a dose of 0.1 mL/kg, 30 min before administering the test substance. To each beagle dog, a formulation corresponding to 20 mg of JIN-001 was administered, and 10 mL of water was provided. All beagle dogs were fed 4 h after drug administration. After drug administration, there was a 7-day washout period, and the second and third administrations were performed in a cross-over design. Blood samples were collected at scheduled time points (0, 0.25, 0.5, 0.75, 1, 1.5, 2, 3, 4, 6, 8, 10, 12, 24, 36, 48, 60 and 72 h). The collected samples were centrifuged at 3000 rpm for 5 min to separate plasma and stored in a cryogenic freezer. Plasma samples were added with an internal standard and subjected to protein precipitation and extraction. JIN-001 concentrations in plasma samples were determined using reversed-phase HPLC equipped with a Turbo Ion Spray MS/MS detector. The cation (M+H)^+^ was analyzed for JIN-001 and betaxolol (IS) in MRM mode.

### 3.10. Statistical Analysis

Data were expressed as mean ±SD, and the mean values between the groups were compared using single-factor analysis of variance. Differences were considered statistically significant when *p* ≤ 0.05. All statistical analyses were performed using Minitab^®^ (ver. 18, Minitab Inc., State College, PA, USA).

## 4. Conclusions

In this study, a JLS tablet with significantly improved solubility and dissolution rate was successfully developed using Capryol 90, a non-volatile liquid vehicle, and CSD, an oil adsorbent. JIN-001 is a promising novel Hsp90 inhibitor for the treatment of lung cancer. From the result of preformulation, it was seen to be poorly soluble with high crystallinity and very low solubility in an aqueous solution. Capryol 90, which was selected based on the oil solubility test, had an excellent solubilizing ability for JIN-001; moreover, JLS powder with good flowability was manufactured due to the excellent adsorption capacity of CSD. The prepared JLS powder showed a significant increase in solubility regardless of pH. As confirmed by the SEM, DSC, and PXRD studies, JIN-001 was dispersed in the CSD in an amorphous form with increased surface area. JIN-001 tablets of various compositions were prepared using excipients with good compatibility between the API and excipients. The JLS tablet showed a significant improvement in the drug release rate, exhibiting a dissolution rate of 80% or more after 30 min in all tested solutions, and it was confirmed that the dissolution rate was higher compared to other JIN-001 formulations; however, in the pharmacokinetic study evaluated in beagle dogs, it was confirmed that the liquisolid system of this study had no significant effect on the bioavailability of JIN-001. The JIN-001 liquisolid tablet proposed in this study is recommended for oral administration with improved solubility and dissolution rate and easy expansion to an industrial scale due to simple manufacturing method. In addition, the solubility and dissolution rate of various poorly soluble drugs can be improved by applying the liquisolid preparation method reported in this study.

## Figures and Tables

**Table 1:** Physicochemical properties of JIN-001 by experimental analysis.

Description	White Powder	Crystalline Structure	Crystalline
Melting point	180.58 °C	Thermal stability	Decomposition after 180 °C
Hygroscopicity	Class I Non-hygroscopic	pKa	Base: 3.30, Acid: 8.86 and 11.13
Bulk density	0.495 g/mL	Tapped density	0.589 g/mL
Carr’s index	16.0%	Hausner ratio	1.19
Log P	3.32	Water solubility	0.04 mg/mL
Log D	pH 1.0: 1.02 pH 1.2: 1.22 pH 2.0: 2.00 pH 3.0: 2.84 pH 4.0: 3.24	pH 5.0: 3.31 pH 6.0: 3.32 pH 6.5: 3.32 pH 7.0: 3.31 pH 7.4: 3.30	pH 8.0: 3.23 pH 9.0: 2.81 pH 10.0: 1.97 pH 11.0: 1.08 pH 12.0: 0.19

**Table 2:** The solubility of JIN-001 in various solvents.

Solvent	Solubility of JIN-001	
	Apparent	Equilibrium (mg/mL)
Water	+	0.04
Ethanol	++++++	68.27
Methanol	++++++	140.02
Acetonitrile	++++	13.56
pH 1.2	+++	9.01
pH 2.0	+	0.70
pH 3.0	+	0.10
pH 4.0	+	0.08
pH 5.0	+	0.06
pH 6.0	+	0.05
pH 6.8	+	0.05
pH 7.0	+	0.06
pH 8.0	+	0.06
pH 9.0	+	0.08
pH 10.0	+	0.10
pH 11.0	+	0.10
pH 12.0	+++	0.94

+: Practically insoluble, +++: Slightly soluble, ++++: Sparingly soluble, ++++++: Freely soluble.

**Table 3:** The apparent solubility of JIN-001 in various oils.

Oil	Apparent Solubility of JIN-001 (mg/mL)	
Soybean oil	++	1 or less
Tween 80	+++	1.7
Labrafil M 1944 CS	+++	2.3
Capryol 90	++++	25.0
Transcutol P	+++++	50.5
Lauroglycol 90	++++	10.2
Labrasol	++++	10.1
Labrafil M 2125 CS	+++	5.6
Labrafac PG	+++	2.8
Labrafac Lipophile WL 1349	+++	1.5
Plurol oleique CC 497	++	1 or less
DL-α-Tocopheryl acetate	++	1 or less
EPAX 4020 TGN	++	1 or less
EPAX 6000 EE	+++	2.2
EPAX 1050 TG/N non tuna	++	1 or less
VivoMega 4030 TG Premium	++	1 or less

++: Very slightly soluble, +++: Slightly soluble, ++++: Sparingly soluble, +++++: Soluble.

**Table 4:** The results of compatibility study by impurities analysis in HPLC.

Items	Initial (%)	4 Weeks (%)	
		25 °C/60 RH	40 °C/75% RH
API	0.39	0.41	0.42
API + Capryol 90	0.40	0.46	0.53
API + MCC	0.39	0.46	0.43
API + SSG	0.45	0.39	0.41
API + PVP	0.48	0.40	0.42
API + MS	0.38	0.41	0.41
API + CSD	0.42	0.41	0.40
API + SSF	5.61	7.05	5.06
API + SLS	0.42	0.45	0.48
API + Gelucire	0.24	0.32	0.69
API + Poloxamer	0.31	0.58	0.36
API + Citric acid	0.60	0.75	0.52
API + Calcium hydroxide	2.06	2.21	3.40

Abbreviations: API, active pharmaceutical ingredient; SSF, sodium stearyl fumarate.

**Table 5:** Flowability of JLS and F2–6 granules (*n* = 3).

Items	Bulk Density (g/mL)	Tap Density (g/mL)	Carr’s Index (%)	Hausner Ratio
JLS	0.28 ± 0.01	0.36 ± 0.01	22.91 ± 0.60	1.30 ± 0.01
F2	0.31 ± 0.01	0.42 ± 0.01	26.97 ± 0.20	1.36 ± 0.00
F3	0.31 ± 0.01	0.42 ± 0.01	26.72 ± 0.83	1.36 ± 0.02
F4	0.30 ± 0.01	0.40 ± 0.02	25.20 ± 1.58	1.34 ± 0.03
F5	0.30 ± 0.01	0.41 ± 0.01	26.81 ± 1.29	1.37 ± 0.02
F6	0.31 ± 0.01	0.42 ± 0.01	26.89 ± 1.35	1.37 ± 0.03

**Table 6:** Pharmacokinetic parameters of JIN-001 following single oral administration of F1, F2 and F4 formulations (20 mg) to beagle dogs (*n* = 9).

Parameter	F1	F2	F4
t_1/2_	9.22 ± 2.82	11.35 ± 3.16	14.28 ± 6.91
T_max_ (h)	0.47 ± 0.23	0.69 ± 0.51	0.47 ± 0.15
C_max_ (ng·h/mL)	9.25 ± 1.98	8.92 ± 2.75	11.29 ± 4.47
AUC_0–48h_ (ng·h/mL)	67.34 ± 25.26	72.07 ± 23.65	64.88 ± 18.57
AUC_infinity_ (ng·h/mL)	71.25 ± 26.50	78.48 ± 25.64	82.37 ± 28.79

**Table 7:** Composition of JIN-001 formulations (mg).

Ingredient	F1	F2	F3	F4	F5	F6
JIN-001	5.0	5.0	5.0	5.0	5.0	5.0
MCC	-	127.0	469.0	350.0	350.0	350.0
PVP	-	7.5	30.0	30.0	30.0	30.0
SSG	-	7.5	30.0	30.0	30.0	30.0
CSD	-	-	60.0	60.0	60.0	60.0
SLS	-	1.5	-	-	-	-
Capryol 90	125.0	-	-	125.0	-	-
Gelucire	-	-	-	-	125.0	-
Poloxamer	-	-	-	-	-	125.0
MS	-	1.5	6.0	6.0	6.0	6.0
Total weight	130.0	150.0	600.0	600.0	600.0	600.0

Abbreviations: MCC, microcrystalline cellulose; PVP, polyvinylpyrrolidone; SSG, sodium starch glycolate; CSD, colloidal silicon dioxide; SLS, Sodium lauryl sulfate; MS, magnesium stearate.

## Data Availability

Data is contained within the article.
